# *Brucella* Cyclic β-1,2-Glucan Plays a Critical Role in the Induction of Splenomegaly in Mice

**DOI:** 10.1371/journal.pone.0101279

**Published:** 2014-07-01

**Authors:** Mara S. Roset, Andrés E. Ibañez, Job Alves de Souza Filho, Juan M. Spera, Leonardo Minatel, Sergio C. Oliveira, Guillermo H. Giambartolomei, Juliana Cassataro, Gabriel Briones

**Affiliations:** 1 Instituto de Investigaciones Biotecnológicas “Rodolfo Ugalde” - Instituto Tecnológico de Chascomús (IIB-INTECH), Universidad Nacional de San Martín (UNSAM), Consejo Nacional de Investigaciones Científicas y Técnicas (CONICET), Buenos Aires, Argentina; 2 Laboratorio de Inmunogenética, INIGEM-CONICET, Hospital de Clínicas “José de San Martín,” Facultad de Medicina, Universidad de Buenos Aires (UBA), Buenos Aires, Argentina; 3 Department of Biochemistry and Immunology, Institute of Biological Sciences, Federal University of Minas Gerais, Belo Horizonte, Minas Gerais, Brazil; Universite de la Mediterranee, France

## Abstract

*Brucella*, the etiological agent of animal and human brucellosis, is a bacterium with the capacity to modulate the inflammatory response. Cyclic β-1,2-glucan (CβG) is a virulence factor key for the pathogenesis of *Brucella* as it is involved in the intracellular life cycle of the bacteria. Using comparative studies with different CβG mutants of *Brucella*, *cgs* (CβG synthase), *cgt* (CβG transporter) and *cgm* (CβG modifier), we have identified different roles for this polysaccharide in *Brucella*. While anionic CβG is required for bacterial growth in low osmolarity conditions, the sole requirement for a successful *Brucella* interaction with mammalian host is its transport to periplasmic space. Our results uncover a new role for CβG in promoting splenomegaly in mice. We showed that CβG-dependent spleen inflammation is the consequence of massive cell recruitment (monocytes, dendritics cells and neutrophils) due to the induction of pro-inflammatory cytokines such as IL-12 and TNF-α and also that the reduced splenomegaly response observed with the *cgs* mutant is not the consequence of changes in expression levels of the characterized *Brucella* PAMPs LPS, flagellin or OMP16/19. Complementation of *cgs* mutant with purified CβG increased significantly spleen inflammation response suggesting a direct role for this polysaccharide.

## Introduction

*Brucella* is a bacterial pathogen that infects ruminants as the primary host and can be transmitted to humans by consumption of animal-derived contaminated products (e.g. unpasteurized dairy food) leading to brucellosis, a reticular endothelial disease, typically characterized by undulant fever [1]. *Brucella*, as the result of a longstanding association with the mammalian host, has evolved modified PAMPs (pathogen-associated molecular patterns molecules) such LPS and flagellin. These modified PAMPs limit host recognition by innate immune receptors (TLRs and NLRs) during the acute phase of the infectious process, reducing the induction of the inflammatory response known to be necessary for an efficient control of the infection [2]–[4]. In addition *Brucella* expresses virulence factors that actively interfere with the innate immune response such as the *Brucella* proteins Btp-A/TcpB and BtpB that down-regulate TLR2/TLR4 signaling [5]–[7]. Paradoxically, when the host is persistently infected at the chronic phase of brucellosis, inflammation becomes a dominant clinical sign that has been described to affect several organs producing symptoms such as arthritis, endocarditis, meningitis, epididymitis and splenomegaly [8].

To date few virulence mechanisms have been described in *Brucella* infection and different genomic studies have confirmed the absence of classical virulence factors like fimbriae, pilli, toxins or plasmids [9], [10]. In our laboratory, we have identified and characterized a critical molecule for *Brucella* virulence: the cyclic β-1,2-glucan (CβG). CβG is composed by a family of cyclic polymers of 17 to 25 D-glucose molecules linked by β-1,2 linkages, synthesized by a membrane bound enzyme named Cgs (for cyclic glucan synthase) that utilizes UDP-glucose as the sugar donor [11]. Cgs initiates and elongates a linear chain of glucoses covalently bound to the Cgs which is subsequently cyclated and released to the bacterial cytoplasm [12]. A specific *Brucella* ABC-transporter Cgt (for Cyclic glucan transporter) translocates the CβG to the periplasmic space where they become chemically modified with the addition of succinyl groups, a reaction catalyzed by the membrane enzyme Cgm (for Cyclic glucan modifier) [13], [14]. Although CβG is present within the periplasmic space, being this localization critical for hypo-osmotic adaptation, secretion of large amount of this polysaccharide to the supernatant by a non-characterized mechanism has been described in *Agrobacterium* and *Sinohizobium* [15]. Interestingly, *Brucella cgs* mutant strain presents a defect in intracellular trafficking in epithelial cells that can be complemented by the addition of purified CβG (or Cyclodextrins) to tissue culture medium [16]. This observation suggests that also in *Brucella*, CβG must be secreted within the host cell to exert its role in virulence. The proposed mechanism of action for CβG in *Brucella* infection is the sequestration of cholesterol from intracellular host membranes leading to lipid raft disorganization and modulation of intracellular trafficking [16].

We have previously observed that mice infected with a *cgs* mutant had a reduced spleen inflammatory response even though they had a high number of replicating bacteria within this organ [17]. Since inflammation is the consequence of the host recognition of microbial PAMPs that ultimately lead to the induction of an inflammatory process, we hypothesized that either the periplasmic CβG may be stabilizing the expression of *Brucella* PAMPs such as LPS, flagellin, and OMPs or that CβG could be recognized by the innate immune receptors in the context of *Brucella* infection triggering inflammation.

It has been shown recently, using *in vitro* studies and purified CβG, that this molecule acts directly as an agonist of the innate immune system mediated by TLR4 in a MyD88/TRIF dependent fashion (and in a CD14 independent manner) [18]. Here we describe the role of *Brucella* CβG in splenomegaly and inflammation.

## Materials and Methods

### Ethics statement

The protocol of this study (reference number 10/2011) was approved by the Committee on the Ethics of Animal Experiments of the Universidad Nacional de San Martín, which also approved protocol development under the recommendations in the Guide for the Care and Use of Laboratory Animals of the National Institutes of Health.

### Growth of *B. abortus*

Bacterial strains used in this study were: *Brucella abortus* strain 2308, *Brucella abortus* strain S19, *Brucella abortus cgs*08 mutant, *Brucella abortus cgs*19 mutant [17]; *Brucella abortus cgt19* mutant [13] and *Brucella abortus cgm19* mutant [14]. All experiments involving live *B. abortus* were conducted in a biosafety level 3 (BSL3) facilities at the University of San Martín, Buenos Aires, Argentina. *B. abortus* strains were grown at 37°C on tryptic soy agar (TSA) and in tryptic soy broth (TSB) on a rotary shaker (250 rpm). If necessary, culture media were supplemented with the appropriate antibiotics at the following concentrations: kanamycin (Km), 50 µg/ml; ampicillin (Amp), 100 µg/ml; and nalidixic acid (Nal), 5 µg/ml.

### CβG purification from *B. abortus*

For preparative purposes, 6-liter of stationary-phase cultures of *B. abortus* S19 grown for 48 h at 37°C (250 rpm) were harvested by centrifugation at 10,000×g for 10 min. The pellets were extracted with ethanol (70% ethanol; 1 h at 37°C). The ethanolic extracts were centrifuged and the supernatants were dried in a speed-vac centrifuge and subjected to Bio-Gel P4 chromatography and HPLC on a C18 silica column for further purification as described previously [14]. Purity of CβG was confirmed by NMR analysis [14]. Thin-layer chromatography (TLC) was performed as previously describe [12].

### Western blot analysis

To monitor the expression levels of outer membrane proteins and LPS in *B. abortus* strains, bacteria were grown in TSB and harvested at stationary phase. Equivalent bacterial pellets were resuspended in Laemmli buffer and samples were subjected to SDS-PAGE. Proteins were transferred onto nitrocellulose membranes using a semi-dry transfer procedure. Immunoblotting was performed using mouse monoclonal antibodies against Omp16 and Omp19 (kindly provided by Dr. Axel Cloeckaert) and mouse anti-O-polysaccharide monoclonal antibody M84 (kindly provided by Dr. K Nielsen). The correct O-antigen display on the membrane of *B. abortus* was confirmed by a *Brucella* phage sensitivity test (Tb^s^, Rc^r^) and crystal violet staining [30].

In order to detect the expression of flagellin in *B. abortus* strains, bacteria were grown in 2YT medium [19] and harvested at early log phase (OD_600_ 0.2). Equivalent bacterial pellets were resuspended in Laemmli buffer and samples were subjected to 12% SDS-PAGE. Proteins were transferred as described above. Immunoblotting was performed using rabbit polyclonal antibodies against *Brucella* flagellin (kindly provided by Dr. J J Letteson).

### Effect of the osmolarity on *B. abortus* growth

All strains were grown in regular LB (170 mM NaCl) until stationary phase. Cultures were harvested by centrifugation (12,500×*g* for 5 min) and washed twice with PBS. Cells were suspended to an OD_600_ of 0.9 and serially diluted in PBS, and 10 µl of the dilutions were spotted on LB agar or LB agar without the addition of NaCl. The plates were incubated at 37°C for 5 days before being read.

### *B. abortus* virulence in mice

Virulence was determined by quantitating *Brucella* survival in mouse spleens at two weeks postinfection, as previously described [20]. Groups of 9-week-old female BALB/c mice were intraperitoneally (i.p) injected with different doses of *B. abortus* strains in 0.2 ml of sterile PBS. The animals were euthanized, and the spleens were removed, weighed, homogenized in PBS, serially diluted, and plated onto TSA with the appropriate antibiotics to determine the number of CFU per spleen.

### Complementation of *B. abortus cgs* mutant with purified CβG

Mice were i.p inoculated with 1×10^6^ CFU of *B. abortus* S19 or 1×10^6^ CFU of its isogenic strain *B. abortus cgs* mutant. Sets of five mice inoculated with *B. abortus cgs* mutant were i.p injected with 15 µg of purified CβG during the first five days of infection. At two weeks postinfection, the animals were euthanized, and the spleens were removed, weighed, homogenized in PBS, serially diluted, and plated onto TSA with the appropriate antibiotics to determine the number of CFU per spleen.

### Histological analysis of spleens infected with *B. abortus*

Group of mice were i.p infected with 1×10^6^ CFU of *B. abortus* S19 or its isogenic *B. abortus cgs* mutant strain. At two weeks postinfection spleens were excised, fixed with 10% neutral buffered formalin and paraffin embedded. Finally, four micrometers thick longitudinal sections of spleens were obtained and stained with hematoxylin and eosin to assess the degree of spleen inflammation.

### Preparation of single-cell suspensions of spleens infected with *B. abortus*

Spleens were aseptically removed and single cell suspensions were prepared by gently teasing through a sterile stainless steel screen. Erythrocytes were lysed in red blood cell lysis buffer and cells were washed twice in PBS solution.

### Determination of inflammatory cell recruitment in spleens infected with *B. abortus*

BALB/c mice were infected with 1×10^6^ CFU of *B. abortus* S19 or its isogenic *B. abortus cgs* mutant strain. Spleens were obtained at two weeks postinfection and single cell suspensions were prepared. To assess recruitment of different cell subtypes after infection, splenocytes (2×10^6^) were stained with anti-CD4 (FITC), -CD8 (PE-Cy5), -CD11c (PE), -CD11b (PE-Cy7), -Ly6G (PE), -Ly6C (PerCP-Cy5.5) and -B220 (PE) and analyzed by flow cytometry using FACSAriaII (BD Biosciences, San Jose, CA) and FlowJo software (Tree Star, Ashland, OR). Monoclonal antibodies were purchased from eBioscience (San Diego, CA) and BD Biosciences (San Jose, CA).

### Determination of cytokines in bone-marrow derived macrophages (BMDM) infected with *B. abortus* strains

Macrophages were derived from bone marrow of C57BL/6, Mal/tirap, TLR4, TLR6 and TLR9 KO mice as follows. Each femur and tibia was flushed with 5 ml of Hank's balanced salt solution (HBSS). The resulting cell suspension was centrifuged, and the cells were resuspended in Dulbecco's modified Eagle's medium (DMEM; Gibco, Grand Island, NY) containing 10% fetal bovine Serum (FBS; Gibco), 1% penicillin/streptomycin (100 µg/mL) and 10% L929 cell-conditioned medium (LCCM) as a source of macrophage colony-stimulating factor (M-CSF). The cells were distributed in 24-well plates and incubated at 37°C in a 5% CO_2_ atmosphere. Three days after seeding, another 0.1 ml of LCCM was added. On the seventh day, the medium was renewed. On the 10th day of culture, the cells were completely differentiated into macrophages [21]. The BMDM were infected with *B. abortus* S19 or its isogenic *B. abortus cgs* mutant strain, corresponding to a multiplicity of infection (MOI) of 100∶1. After 60 min incubation with the bacteria, wells were washed three times with phosphate-buffered saline (PBS) and incubated with fresh medium containing 50 µg ml−1 Gm and 100 µg ml−1 streptomycin to kill non-internalized bacteria After 24 h postinfection, the level of IL-12 (p40) and TNFα in the supernatants of BMDM were measured by ELISA Duoset kit (R&D, Minneapolis, MN). At 4, 24 and 48 hours postinfection, infected C57BL/6 BMDM were washed three times with PBS and lysed with 500 µl 0.1% Triton X-100 (Sigma-Aldrich Co.). The intracellular CFU was determined by plating serial dilutions on TSA with the appropriated antibiotic.

## Results and Discussion

### Transport of *Brucella* CβG to periplasm is required for bacterial-host interaction

**Figure 1:**
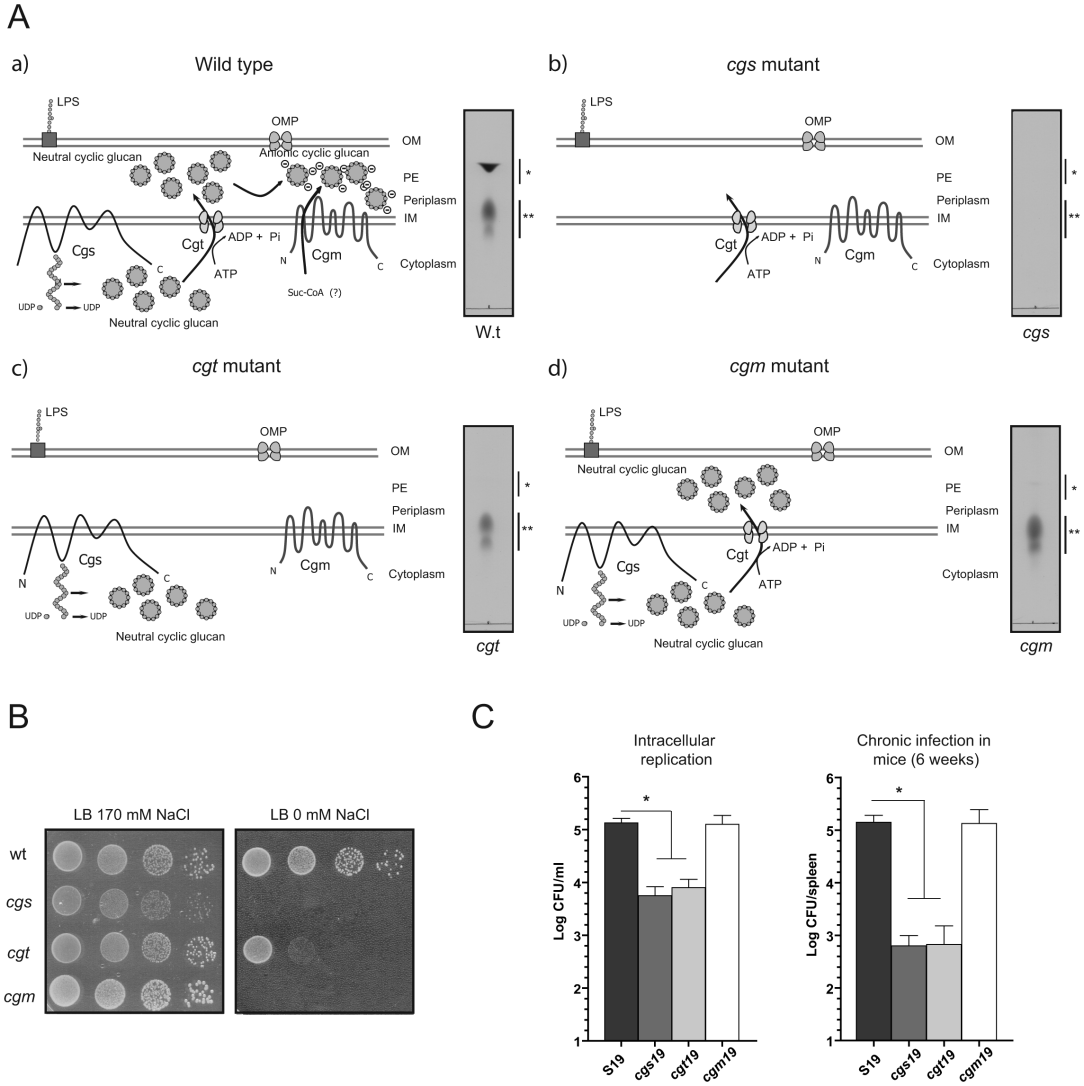
Different roles for *Brucella* CβG. (A) Schematic representation of *Brucella* CβG biosynthesis in the wild type strain and its isogenics CβG mutants strains, *cgs*, *cgt* and *cgm*. Inset pictures show TLC analysis of neutral (**) and anionic (*) CβG. Cgs: Cyclic β-1,2-glucan synthase, Cgt: Cyclic β-1,2-glucan transporter, Cgm: Cyclic β-1,2-glucan modifier. IM: Inner membrane, OM: outer membrane, OMP: outer membrane protein, UDP-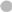: uridine diphospho-glucose, LPS: Lipopolysaccharide. (B) Effect of osmolarity on growth of different strains of *B. abortus*. Dilutions were spotted on LB agar (170 mM NaCl) or LB agar without the addition of NaCl. The plates were incubated at 37°C for 5 days before determining CFU. (C) CβG mutant strains phenotype during *Brucella*-host interaction. Intracellular replication was monitored in HeLa cells at 48 h p.i and establishment of chronic infection of *Brucella* was evaluated in BALB/c mice by determining spleen CFU at six weeks p.i.

As shown in Fig. 1A–a, Cgs, a 320 kDa membrane bound enzyme (the second largest protein in *Brucella*), is the enzyme responsible for the synthesis of cytoplasmic CβG, which is afterwards translocated to the periplasmic space by Cgt, a CβG-specific ABC transporter, and modified with succynil groups by the activity of Cgm (Fig. 1A–a). In order to determine if the different biosynthetic intermediate states of CβG play differential roles in the bacteria the phenotype of three different *B. abortus* CβG mutants were compared: *cgs* [17], *cgt* [13] and *cgm* mutant [14]. As shown in Fig. 1A–b, deletion of *cgs* gene abolished the presence of CβG but mutations in *cgt* or *cgm* lead to the production of neutral CβG (Fig. 1A–c and d) with a different cellular localization being cytoplasmic in *cgt* mutant and periplasmic in the case of *cgm*. From the comparison between *cgt* and *cgm* it became evident that CβG plays at least two roles: adaptation to hypo-osmotic growth conditions (Fig. 1B) and *Brucella*-host interaction (Fig. 1C). While the adaptation to hypo-osmotic conditions requires the presence of anionic periplasmic CβG (Fig. 1B), the sole requirement for host-pathogen interaction is the transport of CβG to the periplasmic space regardless of its anionic charge, as demonstrated by the *cgm* phenotype in intracellular replication and chronic infection in mice (Fig. 1C).

### *B. abortus* mutants unable to produce CβG or to transport it to the periplasm elicit a reduced splenomegaly in mice

**Figure 2:**
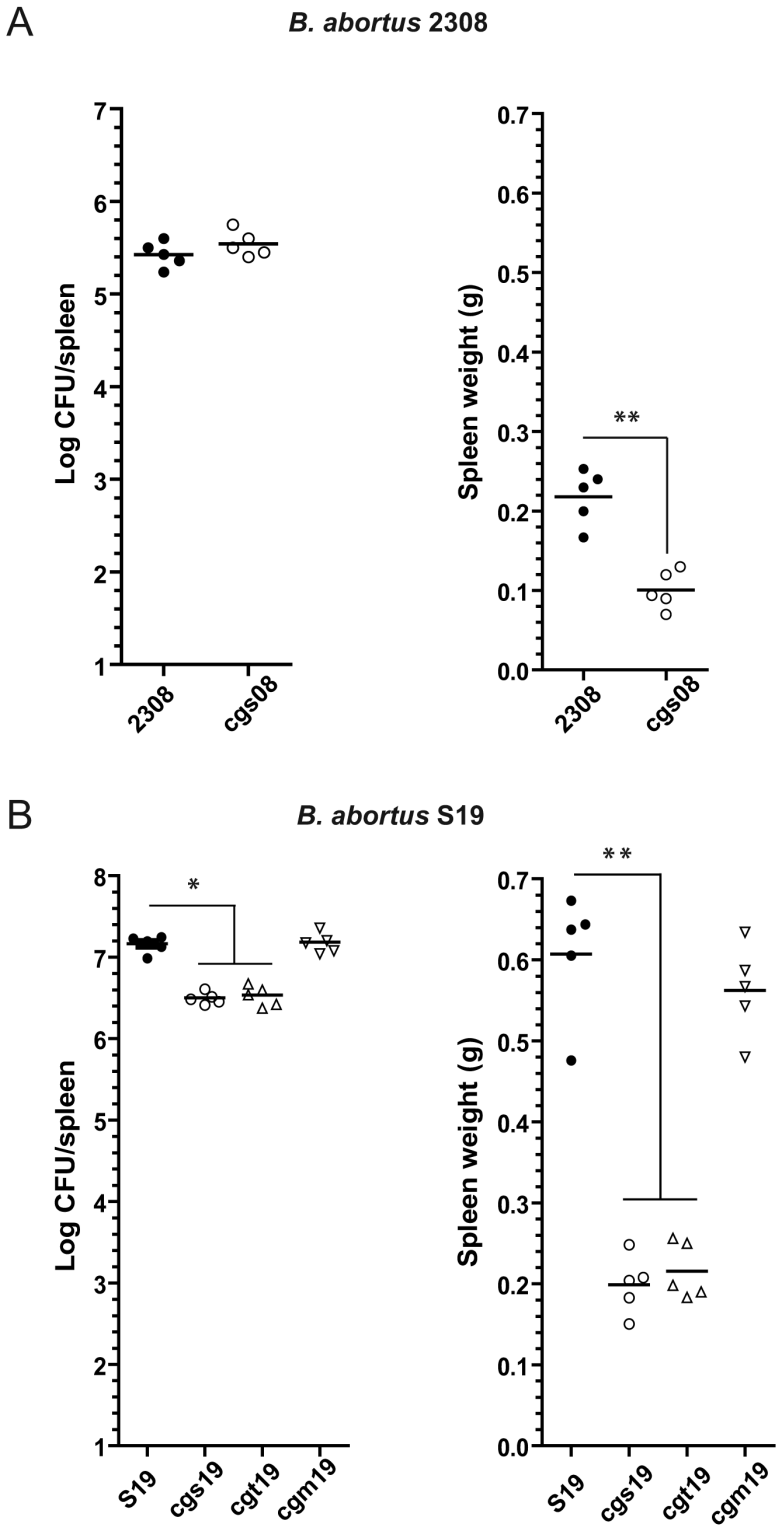
*B. abortus cgs* and *cgt* mutants elicit a reduced splenomegaly in mice. BALB/c mice were intraperitoneally infected (1×10^6^ CFU) with (A) *B. abortus* 2308 or (B) *B. abortus* S19 and their isogenic CβG mutant strains. At two weeks postinfection, spleens were removed, weighed (right panel) and the numbers of CFU recovered were determined by serial dilutions and plating onto TSA (left panel). Five animals were used for each determination. *, *P*<0.05; **, *P*<0.01, Mann-Whitney test.

As we reported, deletion of *cgs* gene either in the *wild type* strain *B. abortus* 2308 or in the vaccine strain *B. abortus* S19 (which is virulent for humans) reduced their ability to infect mice and hampered their efficiency to reach the intracellular replication niche in epithelial cells [16], [17]. Although attenuation of virulence occurs in both *Brucella* backgrounds, in the S19 strain the phenotype is already evidenced after 4 weeks postinfection while in the 2308 background, attenuation is observed after 12 weeks postinfection [17]. Interestingly, at two weeks postinfection when the infection is still not resolved and the number of bacteria in the spleens are equivalent (in the case of *B. abortus cgs*08 mutant, Fig. 2A left panel) or slightly reduced (in the case of *B. abortus cgs*19 mutant, Fig. 2B left panel) compared to their respective wild type parental strains, the splenomegaly was significantly reduced in mice infected with both *cgs* isogenic mutants (Fig. 2A and 2B, right panel). In an earlier report, Crawford et al. observed that splenomegaly elicited by *Brucella melitensis* infection (which peaks from two to three weeks postinfection) is dependent on the initial dose of infection rather than on the bacterial burden, concluding that very early events in the *Brucella* infection are the driving force controlling the severity of the inflammatory response in the spleen [22].

Since *cgs* mutants induced a reduced splenomegaly phenotype in both *B. abortus* 2308 and *B. abortus* S19 backgrounds, we decided to use the vaccine strain of *B. abortus* S19 for the next set of experiments as the wild type control because it elicited a significantly increased splenomegaly compared to the one induced by the *B. abortus* 2308 strain [23] allowing us to develop a more sensitive assay. Additionally, since *B. abortus* S19 presents an intracellular trafficking defect in epithelial cells [24], similar to the defect described for *B. abortus cgs* mutant strains, we reasoned that the use of S19 as our wild type control would reduce also the experimental variability due to differences in intracellular localization. Fig 2B (right panel) shows that the *B. abortus* S19 and *B. abortus cgm19* mutant strains evoked a significantly increased inflammatory response in the spleens in comparison with the mutants *B. abortus cgs19* and *B. abortus cgt19*, suggesting that the splenomegaly correlated with the presence of CβG within the periplasmic space. As mentioned above, CβG is likely secreted within the host cell and therefore periplasmic localization requirement might be potentially a prerequisite for its delivery outside the bacteria.

### Splenomegaly is dependent on the initial dose of *B. abortus* infection

**Figure 3:**
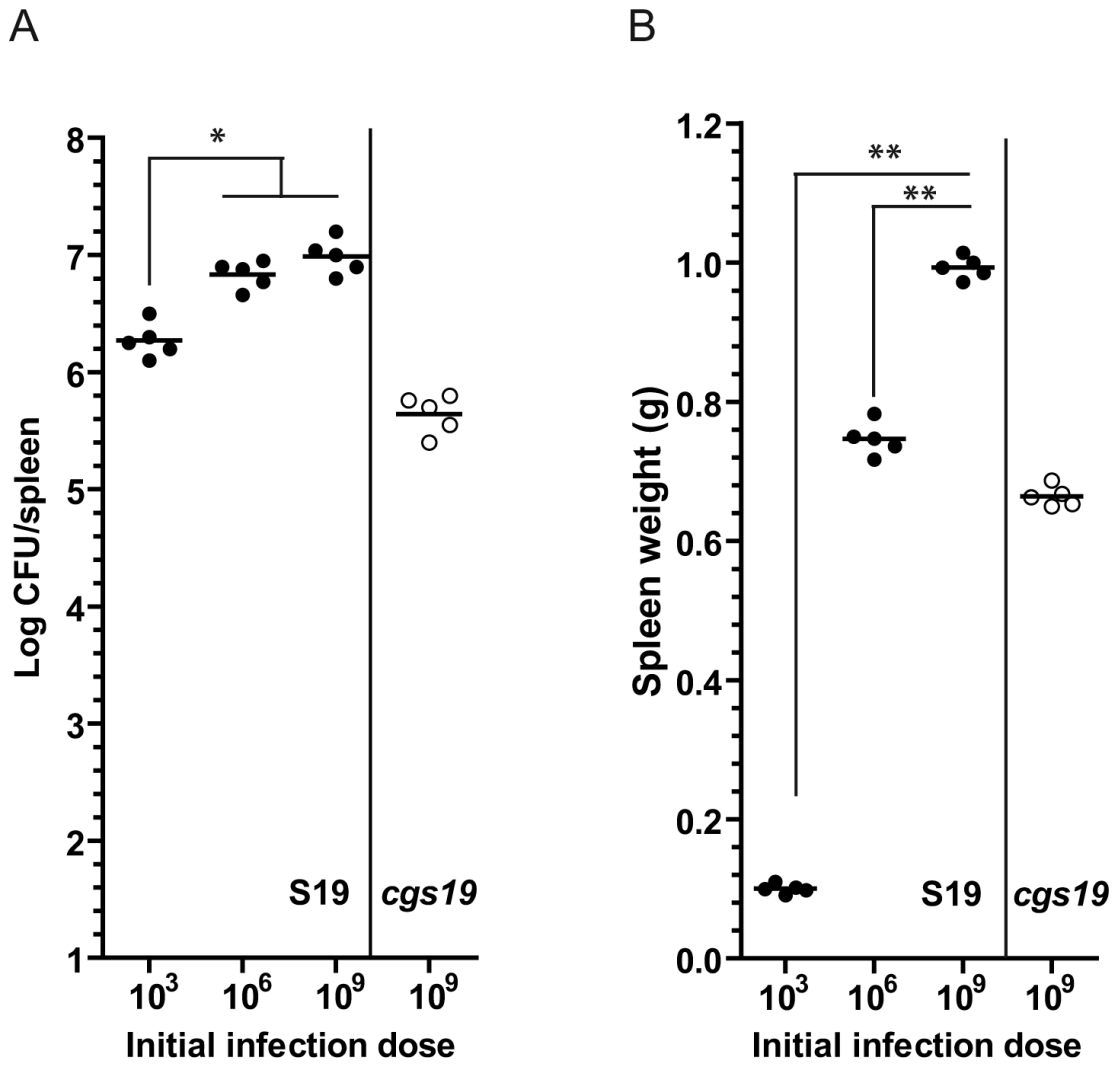
Splenomegaly induced by *B. abortus* is dependent on the initial dose of infection. BALB/c mice were intraperitoneally infected with different doses of *B. abortus* S19 or *B. abortus cgs19* mutant. At two weeks postinfection, spleens were removed, weighed and the numbers of CFU recovered from spleens determined by serial dilutions and plating onto TSA. (A) Recovery of viable bacteria from spleens of mice. (B) Spleens weight of infected mice. Bacterial doses were calculated retrospectively by colony counting. *, *P*<0.05; **, *P*<0.01, Mann-Whitney test.

To study the impact of the initial dose of infection on the intensity of the splenomegaly, BALB/c mice were intraperitoneally infected with different doses of *B. abortus* S19 (10^3^, 10^6^ and 10^9^ CFU) (Fig. 3) and after two weeks postinfection spleens were removed, weighed, homogenized and the number of bacteria determined by serial dilution and plating to determine CFUs. Fig. 3 shows that, at two weeks postinfection although the numbers of replicating *B. abortus* S19 recovered from spleens were similar (about 10^6^–10^7^ CFU per spleen) (Fig. 3A), the splenomegaly varied from negligible (about 0.1 grams) to a massive one (1 gram) (Fig. 3B) depending on the initial dose of infection, confirming previous observations in *Brucella melitensis* [22]. To estimate the impact of *cgs* phenotype on spleen inflammation, we infected mice with different doses of *B. abortus cgs19* mutant strain and at two weeks postinfection, splenomegaly was determined. The results showed that, to achieve an equivalent degree of splenomegaly elicited by 1×10^6^ *B. abortus* S19, it was necessary to increase a thousand times the initial dose of the *B. abortus cgs19* (1×10^9^) (Fig. 3B).

### Reduced splenomegaly elicited by the *cgs* mutant is the consequence of a lesser degree of cell recruitment

**Figure 4:**
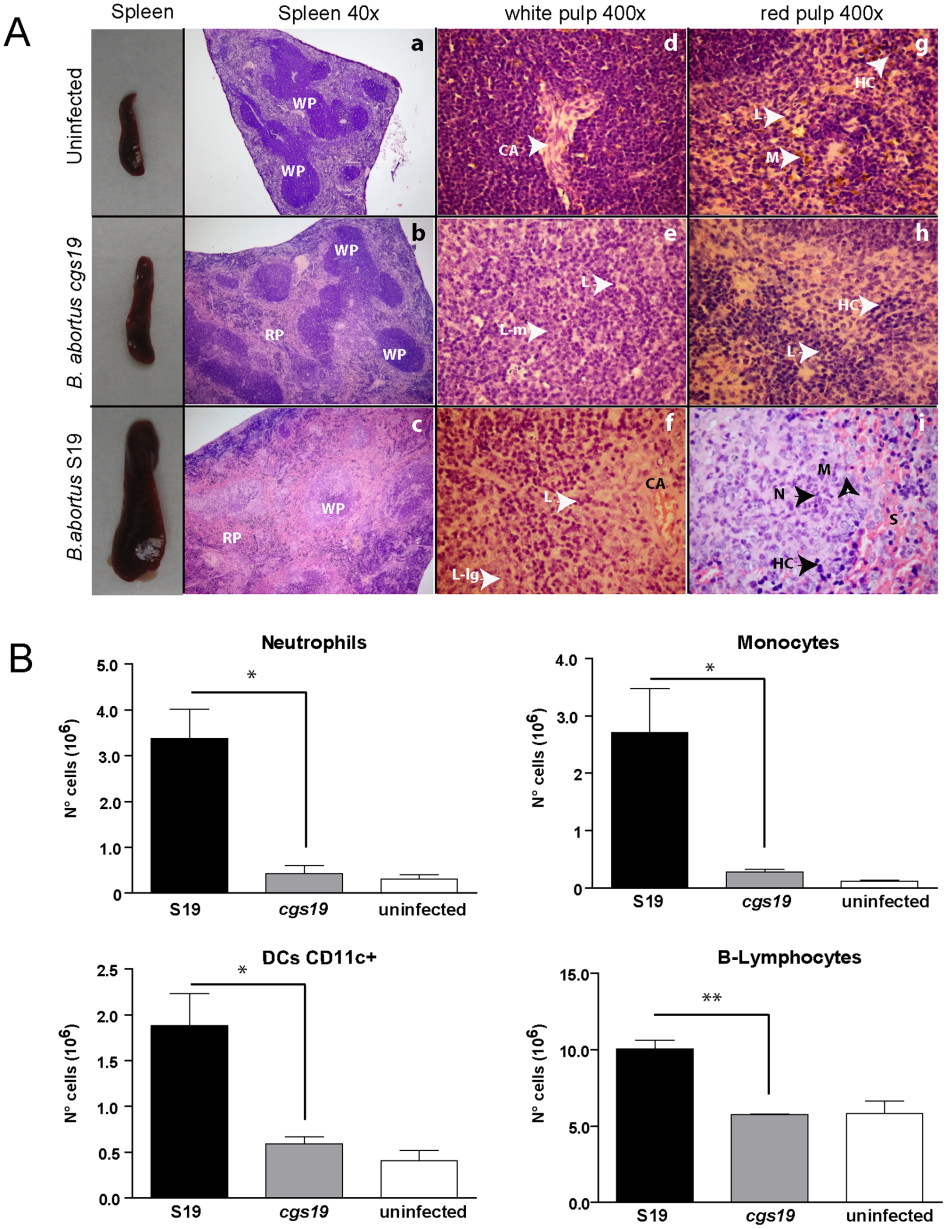
Reduced splenomegaly elicited by *cgs B. abortus* infection is a consequence of a lesser degree of cell recruitment. (A) BALB/c mice were intraperitoneally infected (1×10^6^ CFU) with *B. abortus* S19, *B. abortus cgs19* mutant or PBS as a control. Mice were euthanized at two weeks postinfection and spleens were removed and examined by histological analysis. WP: white pulp; RP: Red pulp; CA: central spleen artery, L: lymphocytes, L-m: medium lymphocytes; L-lg: large lymphocytes, HC: Hematopoietic cells, N: neutrophils, M: macrophages, S: dilated sinuses. (B) The number of differential counts of monocytes, neutrophils, dendritic cells (DCs) and B lymphocytes were determined as described in Materials and Methods. *, *P*<0.05; **, *P*<0.01, *t* test.

At two weeks postinfection spleens from *Brucella* infected mice were processed for histological analysis and the results are shown in Fig 4A. While spleens from wild type strain infected mice showed an increase in the global cellularity with a massive infiltration of the red and white pulp (Fig. 4A–c), spleens from *cgs* infected mice shown a reduced degree of cellular infiltration (Fig. 4A–b) similar to what was observed in spleens of non-infected animals (Fig. 4A–a). Remarkably, at 400× magnification, the presence of neutrophils and macrophages within the red pulp of *B. abortus* S19 infected mice was observed (Fig. 4A–i) indicating massive cell recruitment to the spleen. In addition, spleen white pulp of *B. abortus* S19 and *B. abortus cgs19* infected mice presented reactive lymphocytes (larger and medium lymphocytes) likely due to antigen stimulation (Fig. 4A–e and f).

To further characterize the spleen cell population, antibodies against specific markers were used to identified and quantify neutrophils, monocytes, B cells, T cells and dendritic cells by flow cytometry. As shown in Fig 4B spleens from *B. abortus* S19 infected mice presented eight times more neutrophils, ten times more monocytes, three times more dendritic cells and two times more B cells than spleens infected with *B. abortus cgs19* mutant strain. No difference was observed in T-cell recruitment (not shown).

One possible explanation for the reduced splenomegaly and cell recruitment associated with the lack of CβG biosynthesis or CβG transport to the periplasm could be that *cgs/cgt* strains have a differential expression of PAMPs that might lead to a less efficient engagement of the host innate immune receptors and consequently to a diminished inflammatory response.

### *B. abortus cgs* mutant has normal expression of flagellin and Omps, displaying normal amounts of smooth LPS (S-LPS) on the membrane

It has been demonstrated that *Agrobacterium* and *Sinorhizobium cgs* mutants have a reduced expression of flagellin and a defect in flagella assembly that consequently leads to a non-motile phenotype [25] [26]. Since bacterial flagellins are powerful agonists of innate immune receptors, being recognized extracellularly by the surface receptor TLR-5 and intracellularlly by Nod-like receptors (such Ipaf or Naip5) [2], we explored if the *Brucella cgs* mutant has an altered expression of flagellin that might explain the diminished inflammatory response. In *Alphaproteobacteria* (including the *Brucella* genus) flagellin has a modification in the protein domain recognized by the TLR-5 innate immune receptor supporting the idea that *Brucella* flagellin has evolved to escape the host innate immune recognition [2]. In addition, in *Brucella*, flagellin expression is tightly controlled and only expressed under very strict culture conditions [19] and, because it has never been identified in any intracellular *Brucella* proteomic studies, this suggests that it is expressed poorly within the host cell [27], [28]. In order to determine if the absence of periplasmic CβG affects flagellin expression, *B. abortus* S19 and its isogenic *cgs*, *cgt* and *cgm* mutants were grown in the conditional media to allow flagella assembly as described in Materials and Methods. Expression of *Brucella* flagellin was monitored by Western blot analysis, and the results, shown in Fig. S1A, indicates no changes in flagellin expression. Afterwards, we studied the expression of other critical *Brucella* PAMPs such as OMP16, OMP19 [29] or LPS [3], [4] [31] and no differences in expression were observed associated to any *B. abortus* CβG mutant strains (Fig. S1A and S1B).

### Purified *Brucella* CβG partially complemented the splenomegaly defect in *cgs*-infected mice

**Figure 5:**
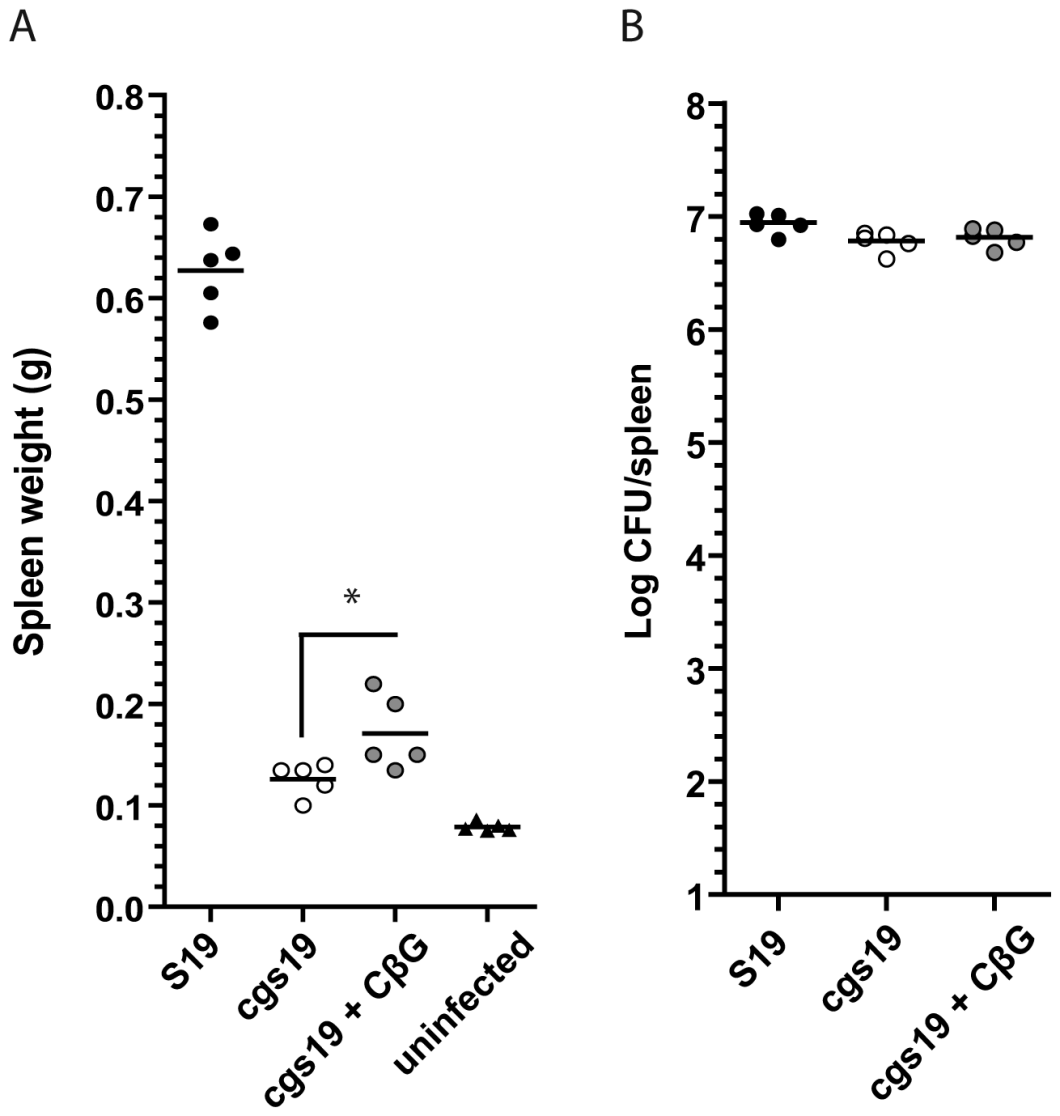
*B. abortus* CβG partially complement splenomegaly in mice. BALB/c mice were intraperitoneally infected with 1×10^6^ CFU of *B. abortus* S19 or 1×10^6^ CFU of *B. abortus cgs19* mutant. Sets of five mice inoculated with *B. abortus cgs* mutant were injected intraperitoneally with 15 µg of purified CβG during the first five days of infection. At two weeks postinfection, mice were euthanized, and spleens were removed, weighed (A) and the number of CFU recovered determined by serial dilutions and plating onto TSA (B). *, *P*<0.05, Mann-Whitney test.

As was mentioned above, purified CβG is capable to restore the intracellular replication deficiency of *cgs*-mutant strain in HeLa cells [16] and this observation suggests that CβG must be secreted within the host cell to exert its role in virulence. To evaluate the direct role of *Brucella* CβG in the inflammatory response we designed a trans-complementation experiment using purified CβG. For this, we added the purified carbohydrate to the bacterial initial inoculum and during the following five days postinfection by i.p injection. Splenomegaly determined at two weeks postinfection showed an increase in spleen weight in the CβG complemented mice compared to the *cgs* mutant although not to the degree of the *Brucella* wild type strain (Fig. 5A). Although significant, trans-complementation with purified CβG elicits a moderate increase on spleen enlargement compared with *cgs* mutant strain, an effect that can be explained as the result of the intrinsic limitations of this experimental approach. For instance, since injected CβG is diluted within the peritoneal cavity is not possible to know the effective CβG concentration at the *Brucella* intracellular replicative niche. However, these results suggest that *Brucella* CβG plays a direct role in the induction of the inflammatory response in the spleen. It has been recently proposed that purified *Brucella* CβG may act as a novel class of adjuvant that can induce, *in vitro*, the activation of mouse and human dendritic cells, enhancing T cell responses and CD4+ T cells memory immune responses in a TLR4 dependent/CD14 independent fashion [18]. Interestingly, it was described recently that inflammation far from be an undesirable byproduct of the bacterial infection can be a process that pathogens can actively promote to create favorable conditions for its own advantage. Thus enterobacterial pathogens can outgrow commensal bacteria or promote host release of compounds of nutritional relevance mediated by the activity of its virulence mechanisms [32], [33]. In that manner it is conceivable to speculate that CβG dependent splenomegaly might work as an advantage for host colonization in *Brucella* infection.

### *B. abortus cgs* mutant strain elicits a reduced inflammatory response in BMDM

**Figure 6:**
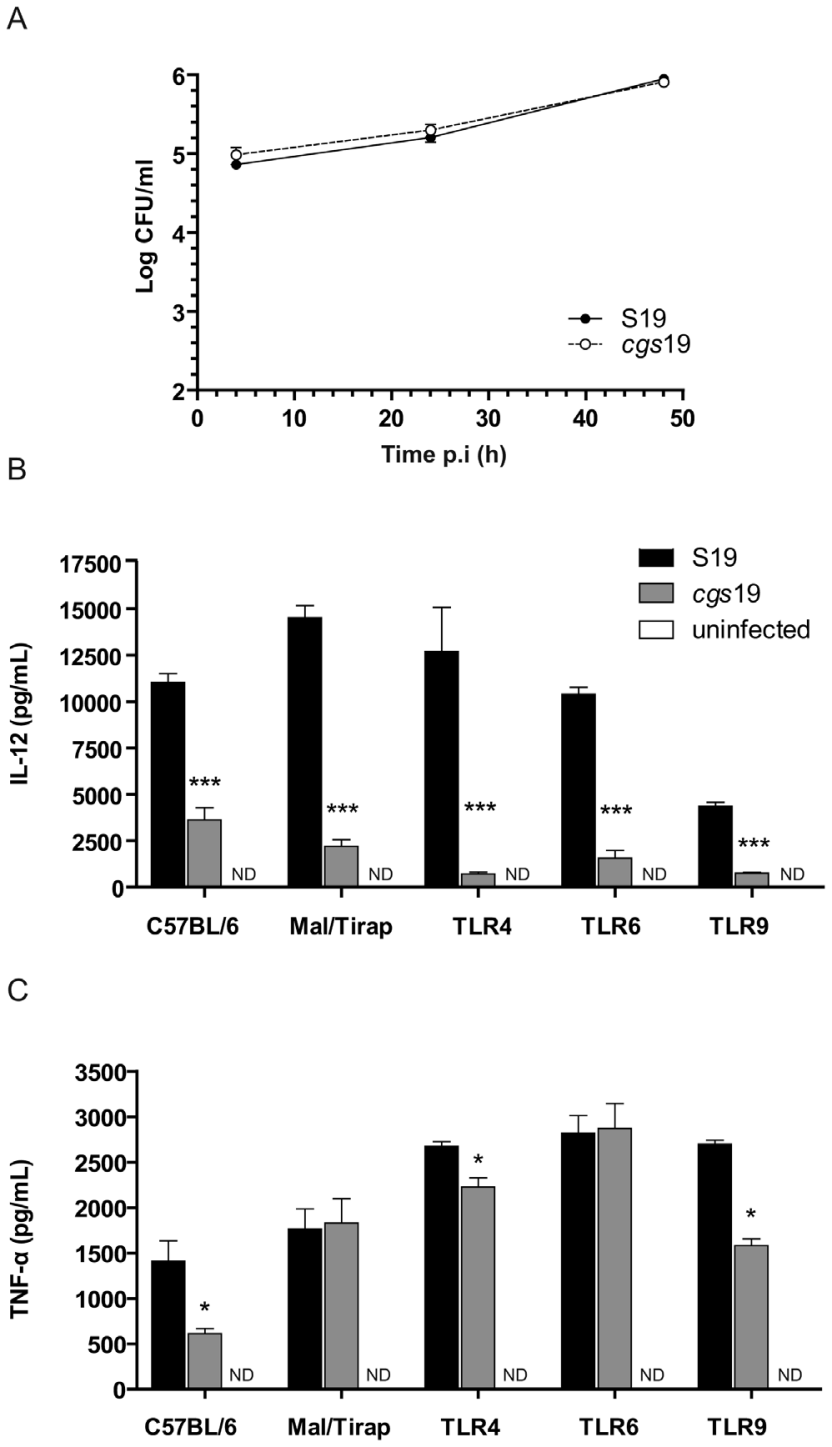
*B. abortus cgs* mutant failed to induce IL-12 in BMDM. (A) Intracellular multiplication of *B. abortus* strains in Bone marrow cells derived from C57BL/6. Number of CFU of intracellular bacteria was determined after lysis of infected cells at the indicated times postinfection. Each determination was performed in duplicate, and values are shown as the means ± standard deviations and are representative of three independent experiments. (B) Bone marrow cells derived from C57BL/6, Mal/Tirap, TLR4, TLR6 or TLR9 KO mice were infected with *B. abortus* (MOI 1∶100). IL-12 and TNF-α were measured by ELISA at 24 h postinfection. *, *P*<0.05; **, *P*<0.01, *** *P*<0.01 *t* test.

In previous studies, Zhan *et al* demonstrated that the macrophage-synthesized cytokines IL-12 and TNF-α are required for an efficient control of *Brucella* infection. In addition, it was shown that depletion of both pro-inflammatory cytokines by antibody treatment abolished the development of splenomegaly in animals infected with *B. abortus* S19 at two weeks postinfection [34]. To understand if the reduced spleen inflammation observed in mice infected with *B. abortus cgs19* was due to a lower induction of IL-12 or TNF-α, we performed an *in vitro* infection experiment with naïve BMDM. Differently to the defective intracellular replication phenotype reported for *B. abortus cgs* mutant strains in HeLa cells [17], in BMDM *B. abortus cgs*19 mutant strain showed no defect in intracellular replication in comparison with its parental wild type strain (Fig. 6A). The same phenotype was also reported for *cgs* mutant strain for intracellular replication in dendritic cells [5]. As shown in Fig. 6B and 6C, wild type BMDM infected with *B. abortus* S19, secreted higher levels of IL-12 (Fig. 6B) and TNF-α (Fig. 6C) to the supernatant than cells infected with *B. abortus cgs*19 mutant strain, suggesting that CβG promotes the induction of proinflammatory cytokines from BMDM. To understand if this CβG-dependent IL-12/TNF-α induction is dependent on Toll-like receptor (TLR) recognition, an *in vitro* experiment with BMDM from Mal/Tirap (the TLR2/TLR4 adapter protein), TLR4, TLR6 and TLR9 KO mice was performed (Fig. 6B and C). As shown in Fig. 6B, CβG-dependent IL-12 induction was independent on the presence of TLR2, TLR4, TLR6 or TLR9 (Fig 6B) while CβG-dependent TNF-α induction was independent on the presence of TLR4 or TLR9 (Fig. 6C). In absence of TLR2 or TLR6, *B. abortus* S19 and its isogenic *cgs* mutant strain elicited similar levels of TNF-α (Fig. 6C). These results suggest that TLR2 and TLR6 are potentially involved in the TNF-α induction elicited by CβG. It is interesting to notice that TLR2 and TLR6 are able to interact to form a heterodimer which is responsible for bacterial deacylated lipoproteins recognition [35].

Taken together all these results suggest that the reduced splenomegaly observed in *cgs* mutant strain infected mice is a consequence of a lower induction of proinflammatory cytokines that lead to a lesser cell recruitment to this organ.

## Concluding Remarks

In the present study we describe the role of the *Brucella* cyclic β-1,2-glucan in promoting spleen enlargement during bacterial infection. Splenomegaly was the result of massive cell recruitment, mediated by the induction of pro-inflammatory cytokines. Since mutants deficient in CβG biosynthesis in the soil bacteria *Sinorhizobium* and *Agrobacterium* have shown to have membrane alterations that lead to non-motile phenotypes and an increased sensitivity to dyes and detergents, we evaluated if the low-inflammation phenotype observed with the *cgs/cgt* mutants was due to changes in expression of membrane bound complexes with inflammatory activity. No differences in flagellin, OMPs or LPS expression were evident and results suggested that CβG per se is responsible for the splenomegaly observed. The molecular mechanism underlying CβG induced splenomegaly remains to be identified and further studies will be performed to characterize this process.

## Supporting Information

Figure S1 **Western blot analysis of flagellin, outer membrane proteins (Omps) (A) and LPS (B) in** ***B. abortus*** **CβG mutant strains.** Immunoblotting was performed using: rabbit polyclonal antibodies against *Brucella* flagellin, monoclonal antibodies against Omp16 and Omp19; and O-antigen specific monoclonal antibody (M84). SDS-PAGE and Western blot were carried out as described in Materials and Methods. The same amount of total protein extracts were loaded into the gels. The estimated molecular weight of each protein is shown.
(TIF)
