# Supplementary figures and images for: Brucella Cyclic β-1,2-Glucan Plays a Critical Role in the Induction of Splenomegaly in Mice
Source: PLoS One. 2014 Jul 1;9(7):e101279. doi: 10.1371/journal.pone.0101279 (PMC4077732; doi:10.1371/journal.pone.0101279)

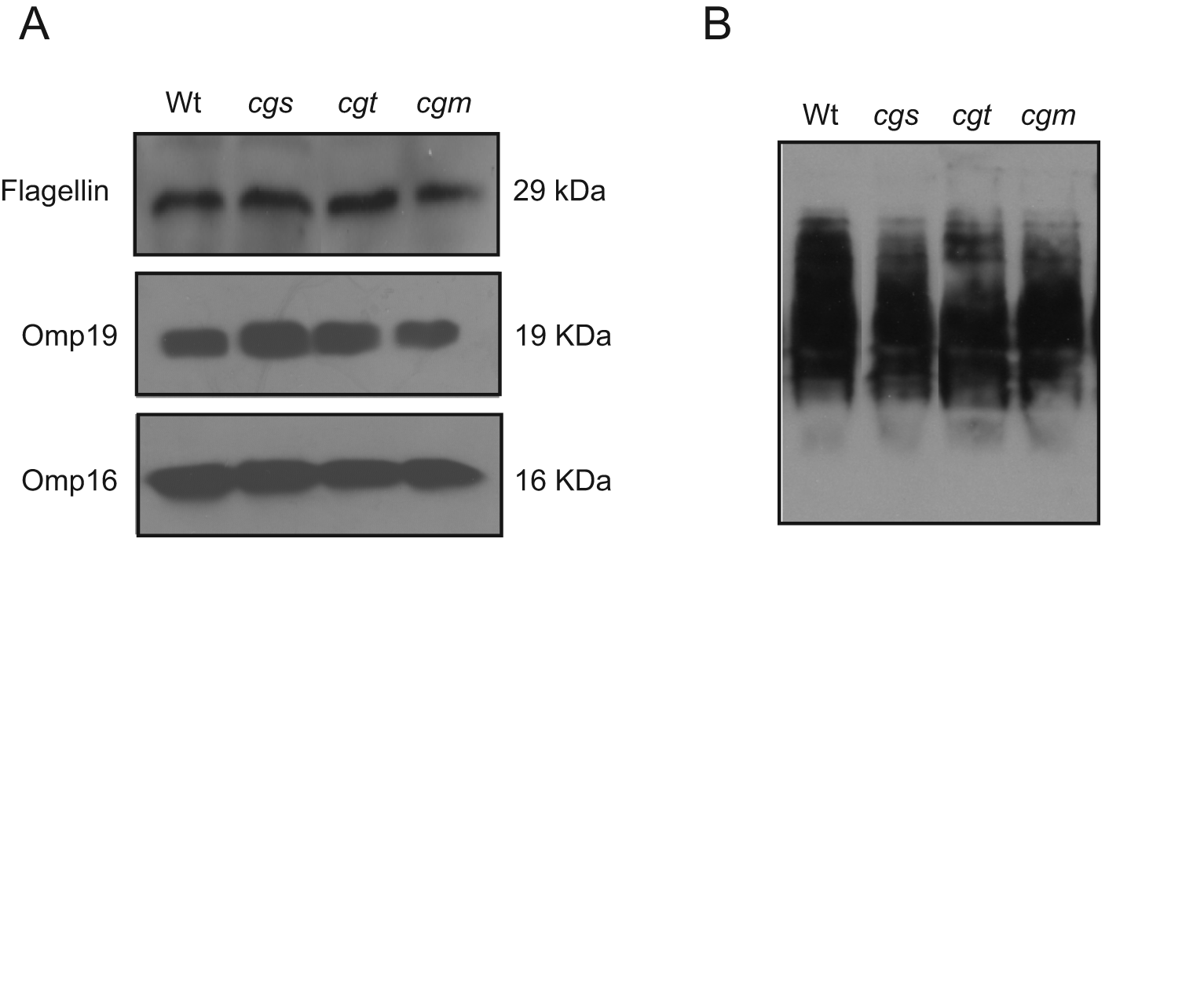

Supplement: Figure S1 — Western blot analysis of flagellin, outer membrane proteins (Omps) (A) and LPS (B) in B. abortus CβG mutant strains. Immunoblotting was performed using: rabbit polyclonal antibodies against Brucella flagellin, monoclonal antibodies against Omp16 and Omp19; and O-antigen specific monoclonal antibody (M84). SDS-PAGE and Western blot were carried out as described in Materials and Methods. The same amount of total protein extracts were loaded into the gels. The estimated molecular weight of each protein is shown. (TIF) [file pone.0101279.s001.tif]
